# The role of MOTS-c-mediated antioxidant defense in aerobic exercise alleviating diabetic myocardial injury

**DOI:** 10.1038/s41598-023-47073-0

**Published:** 2023-11-13

**Authors:** Mi Tang, Quansheng Su, Yimei Duan, Yu Fu, Min Liang, Yanrong Pan, Jinghan Yuan, Manda Wang, Xiaoli Pang, Jiacheng Ma, Ismail Laher, Shunchang Li

**Affiliations:** 1 School of Physical Education, Xihua University, Chengdu, 610039 China; 2 Institute of Sports Medicine and Health, Chengdu Sport University, Chengdu, 610041 China; 3 Department of Anesthesiology, Pharmacology and Therapeutics, Faculty of Medicine, University of British Columbia, Vancouver, BC V6T 1Z3 Canada

**Keywords:** Cardiovascular biology, Cardiovascular biology, Cardiomyopathies

## Abstract

Myocardial remodeling and dysfunction are commonly observed in type 2 diabetes mellitus (T2DM). Aerobic exercise can partly alleviate diabetes-induced myocardial dysfunction through its antioxidant actions. MOTS-c is a potential exercise mimic. This study aimed to investigate the effects of MOTS-c on improving diabetic heart function and its mechanism and to identify whether MOTS-c improved antioxidant defenses due to aerobic exercise. Herein, we established a rat model of T2DM induced by high-fat diet combined with a low-dose streptozotocin injection. Interventions were performed using intraperitoneal injections of MOTS-c (i.p. 0.5 mg/kg/day, 7 days/week) or aerobic exercise training (treadmill, 20 m/min, 60 min/day, 5 days/week) for 8 weeks. Myocardial ultrastructure was assessed using transmission electron microscopy (TEM), myocardial lipid peroxidation levels (MDA), superoxide dismutase (SOD), glutathione (GSH), and catalase (CAT) levels were assessed using colorimetric methods, and molecular analyses including MOTS-c, Kelch-like ECH-associated protein 1 (Keap1), Nuclear factor E2-related factor 2 (Nrf2), adenosine 5'-monophosphate (AMP)-activated protein kinase (AMPK)and phospho-AMPK (p-AMPK) were examined using Western blot. The results showed that MOTS-c, with or without exercise, reduced myocardial ultrastructural damage and improved glucolipid metabolism and cardiac function in T2DM. Furthermore, MOTS-c increased antioxidant markers such as SOD, CAT, and the protein expression of myocardial MOTS-c, Keap1, Nrf2, and p-AMPK. MOTS-c with exercise treatment reduced myocardial MDA and increased p-AMPK significantly comparing to only exercise or MOTS-c alone. Our findings suggest that MOTS-c may be a helpful supplement for overcoming exercise insufficiency and improving myocardial structure and function in diabetes.

## Introduction

Cardiac dysfunction is a major complication of diabetes^1^, where early clinical manifestations include diastolic dysfunction, followed by systolic dysfunction and eventually congestive heart failure^2^. Physical exercise exerts various protective effects on diabetic cardiac insufficiency, including improving cardiomyocyte metabolism, inhibiting cardiomyocyte apoptosis, attenuating myocardial fibrosis, and relieving oxidative stress damage^3,4^. Exercise also prevents myocardial ultrastructural disorders and diastolic dysfunction in diabetic rats and increases Ca^2+^ concentrations in the cardiac sarcoplasmic reticulum and cytoplasm^5^. Additionally, exercise can activate adenosine 5'-monophosphate (AMP)-activated protein kinase (AMPK), a "metabolic master switch" that regulates cellular energy metabolism by detecting and responding to fluctuations in AMP/ adenosine triphosphate (ATP) ratios at rest and during exercise^6,7^. The activation of AMPK by exercise involves a complex mechanism, whereby alterations in energy metabolism and the nuclear accumulation of nuclear factor E2-related factor 2 (Nrf2) activates antioxidant response element (ARE)-driven gene transactivation^8^. Furthermore, exercise increases reactive oxygen species (ROS) levels through various mechanisms and then triggers the activation of Nrf2^9,10^.MOTS-c is an open reading frame peptide encoded by a mitochondrial 12 s rRNA gene^11^, which mainly targets muscle tissue, significantly enhances insulin sensitivity, and improves glucose utilization^12,13^. MOTS-c targets various organs through the circulation, including the lungs^13–16^, spinal cord^17^, skeletal muscles^12,18^, adipose tissues^13,19–23^, brain^19,24^, liver^13,15,21,22,25,26^, bone^20,27–30^, spleen^13,15^, kidney^13,15^, heart^13,15,31–34^, blood vessels^33,35^, skin^22^, and the small intestine^22^. MOT-c is important in regulating dysglycemia and metabolic changes in diabetic patients^36,37^. Lipocalin can act as a regulator of MOTS-c and mediates skeletal muscle MOTS-c production and/or secretion via the adaptor protein, phosphotyrosine interaction, PH domain, and leucine zipper containing 1(APPL1)/ sirtuin 1(SIRT1)/professional generated content-1(PGC-1 α) pathway. In addition, MOTS-c is a potential new target for treating diabetes mellitus^18^. We previouly reported that MOTS-c administration increased endogenous levels of myocardial MOTS-c and activated AMPK, improves myocardial mechanical efficiency, enhanced cardiac contractility, and improved diastolic function during exercise training^32^. Also, MOTS-c intervention could also improve cardiac function in diabetic rats through the cysteinerich61 (CCN1)/ extracellular regulated protein kinases1/2(ERK1/2)/recombinant early growth response protein 1 (EGR1) pathway^38^. Earlier studies have not addressed the effects of combined MOTS-c and exercise interventions on diabetic cardiac function or examined the effects of MOTS-c on diabetic myocardial oxidative stress. We hypothesized that MOTS-c can mimic the effects of exercise by improving cardiac dysfunction in a rat model of T2DM.The underlying mechanism may be that MOTS-c improves the oxidative stress pathway involving Kelch-like ECH-associated protein 1 (Keap1)—Nrf2.

## Materials and methods

### Experimental materials

Sixty-six male Sprague–Dawley (SD) rats (6 weeks old, 170–185 g) were purchased from Chengdu Dashuo Experimental Animal Co., and housed in an animal housing room maintained at 22–25 °C and air humidity of 40–60%. that was well ventilated and had a 12/12-h light/dark cycle. The rats were fed with either standard chow or high-fat and high-sugar chow (67% standard chow, 20% sucrose, 10% lard, 2% cholesterol, and 1% sodium cholate, Chengdu Dashuo Experimental Animal Co., Ltd.). The experiments were completed at the Institute of Sports Medicine and Health in Chengdu Sport University. Our experimental protocol was approved by the Ethics Committee of Chengdu Sport University (Number: 2021-07) and performed in accordance with relevant guidelines and regulations. The reporting of our study follows the recommendations of the animal research: reporting of in vivo experiments (ARRIVE)guidelines^39^.

### Animal model and experimental groups

After 2 weeks of acclimatization, 10 rats were randomly selected for the control group (C) and fed with standard chow, while the other 56 rats were fed the high-fat and high-sugar chow. Blood glucose and insulin levels of rats were measured after 7-weeks of feeding with the high-fat and high-sugar diet, and the insulin resistance index was calculated. Streptozotocin (STZ, 30 mg/kg, Sigma, USA) was injected intraperitoneally in rats fed with high-fat and high-sugar diets, and non-fasting blood glucose (NFBG) levels were measured 72 h later. Levels of NFBG ≥ 16.7 mmol/L indicated the successful establishment T2DM in the rats^40^. The same volume of saline solution (30 mg/kg) was injected intraperitoneally into control chow-fed rats. 40 rats that developed T2DM were randomly divided into four groups (n = 10 per group): diabetic group (D), diabetic exercise group (DE), diabetic MOTS-c group (DM), and diabetic exercise + MOTS-c group (DME).

### Aerobic exercise protocol

Rats in the DE and DME groups were trained on a treadmill exercise protocol based on the Bedford motion model protocol^41^, using a speed of 15 m/min for 3 days for acclimatization, after which the treadmill speed was increased to 20 m/min for 60 min/day. Rats were exercised 5 days/week for 8 weeks.

### MOTS-c intervention

Rats in the DM and DME groups were injected intraperitoneally with MOTS-c (0.5 mg/kg/day, 7 days/week, GL Biochem Shanghai Ltd. China) for lasts 8 weeks^12^, while rats in the C and D groups were injected with normal saline (0.5 mg/kg/day) in the same way and same volume identical to MOTS-c.

### Measurements of blood glucose, insulin and lipids

Blood samples (0.05 ml) were taken from the tail vein of rats, and non-fasting blood glucose (NFBG) and fasting blood glucose (FBG) levels were measured using a blood glucose meter (OneTouch Ultra Vue Johnson & Johnson, New Brunswick, NJ, USA). After 8-weeks of interventions (exercise with and without MOTS-c treatment), blood was collected from the abdominal aorta of rats in each group, which was then centrifuged at 2500 r/min for 10 min to obtain serum, which was stored at − 80 °C for later use. Serum fasting insulin (FINS) was measured by ELISA (Immuno Way Biotechnology Company, USA), and homeostatic model assessment for insulin resistance (HOMA-IR) was calculated as [Plasma glucose (GLU, mmol/L) X serum insulin (m IU/L)]/22.5. A Hitachi 7600 automatic biochemical analyzer was used to measure triglyceride (TG), total cholesterol (TC), high-density lipoprotein cholesterol (HDL-C), and low-density lipoprotein cholesterol (LDL-C) levels.

### Transmission electron microscopy (TEM)

The rat left ventricular myocardium was fixed with 3% glutaraldehyde, refixed with 1% osmium tetroxide, stepwise dehydrated with acetone, followed by Epon812 embedding and polymerization by heating to form an embedding block. Ultrathin sections (~ 50 nm thick) sections were prepared with an ultrathin sectioning machine. The sections were then first stained with uranyl acetate and then with lead citrate. Finally, the ultrastructure of the myocardium was observed by TEM using a transmission electron microscope (JEM-1400PLUS; JEOL, Tokyo, Japan).

### Echocardiography

A GE VIVID 7 cardiac color ultrasound diagnostic instrument with a 10S cardiac probe was used to obtain cardiac images. The precordial region of each rat was first shaved, and 1% pentobarbital sodium (0.15 mL/100 g) injected intraperitoneally. After successful induction of anesthesia, rats were placed in a supine position, and M-mode ultrasound tests were performed to record interventricular septal thickness at diastole (IVSd), left ventricular internal diameter at end-diastole(LVIDd), left ventricular posterior wall thickness at end-diastole(LVPWd), left ventricular internal dimension systole(LVIDs), left ventricular ejection fraction (EF), left ventricular short-axis shortening index (FS%), and early diastolic flow velocity/late diastolic flow velocity ratio (E/A). All measurements were obtained using the average values obtained during three consecutive cardiac cycles. All test indicators were measured using a double-blind method by the same examiner with the same instrument.

### Colorimetry

Myocardial oxidative stress biochemical indexes were analyzed using CheKine™ Superoxide Dismutase (SOD) Activity Assay Kit (Item No. KTB1030, Abbkine, USA.), CheKine™ Catalase (CAT) Activity Assay Kit (Item No. KTB1040, Abbkine, USA), CheKine™ Lipid Peroxidation (MDA) Assay kit (Item No. KTB1050, Abbkine, USA), and CheKine™ Reduced Glutathione (GSH) Colorimetric Assay Kit (Item No. KTB1600, Abbkine, USA).

### Western blotting

Myocardial tissue was lysed in radioimmunoprecipitation assay buffer (RIPA) lysate (AR0102-30, BOSTER, 30 ml) and ethylenediaminetetraacetic acid (EDTA)-containing protease inhibitor (100 ×) buffer before extraction of the protein and was sonicated to fragment the myocardial tissue. A centrifuge was used at 13,000 rpm for 3–5 min at 4 °C to remove cellular debris. The supernatants were analyzed using homemade gels with pre-cast 10% sodium dodecyl sulfate polyacrylamide gel electrophoresis (SDS-PAGE) gels (omni-EasyTM) for electrophoresis. The resolved gels were transferred from the pre-cast SDS-PAGE gels to polyvinylidenefluoride (PVDF) membranes, and blocking was performed with 5% bovine serum albumin (BSA) in Tris-buffered saline (TBS-T) containing 0.05% Tween-20 and incubated with the primary antibodies: MOTS-c antibody (MOTSC-101AP, FabGennix); Keap1 antibody (Keap1 10503-2-AP, Proteintech); Nrf2 antibody (Nrf2 ER1706-41-10, Huabio); Adenosine 5ʹ-Monophosphate (AMP)-activated Protein Kinase (AMPK) antibody (AMPK ET1608-40, Huabio); p-AMPK alpha (Thr172) antibody(p-AMPK AF3423, Affinity) with the concentrations of diluted antibodies shown in Table 1. Diluted primary antibodies were added and incubated overnight at 4 °C. The membranes were washed three times with TBS-T and incubated with the HRP-conjugated secondary antibody for 2 h at room temperature. The membranes were washed three more times, developed with Ultra Signal ultra-sensitive ECL chemiluminescent substrate (Beijing 4A Biotechnology Co., Ltd.), and imaged with the Vision Works system (Analytik Jena AG, Germany). All experiments were repeated three times with three replicates/group/experiment.

**Table 1:** Dilutions of concentrations of antibodies for Western blotting.

Antibody	Predicted molecular weight (kDa)	Source	Primary antibody dilution ratio
MOTS-c	2	Rabbit	1:500
p-AMPK	62	Rabbit	1:1000
β-actin	43	Rabbit	1:5000
Keap1	70	Rabbit	1:2000
Nrf2	68	Rabbit	1:1000
AMPK	63	Rabbit	1:2000
GAPDH	37	Rabbit	1:5000

### Statistical analysis

Statistical analysis was performed using IBM SPSS Statistics 22 software. Quantitative data that approximately obeyed normal distribution is expressed as mean ± standard deviation (mean ± SD). For quantitative data obeying normal distribution, least significance difference (LSD) and Duncan's test of one-way analysis of variance (ANOVA) were used if the variance was homogeneous, and Dunnett's T3 test of one-way ANOVA was utilized to test the difference between multiple groups if the variance was not homogeneous, and the significance level was set at p < 0.05.

### Institutional Review Board Statement

The animal study protocol was approved by the Ethics Committee of Chengdu Sport University (protocol code 2021-07 and date of approval: March, 9, 2021)” for studies involving animals. Animals involved in this study are male SD rats.

## Results

### Fasting blood glucose

**Figure 1:**
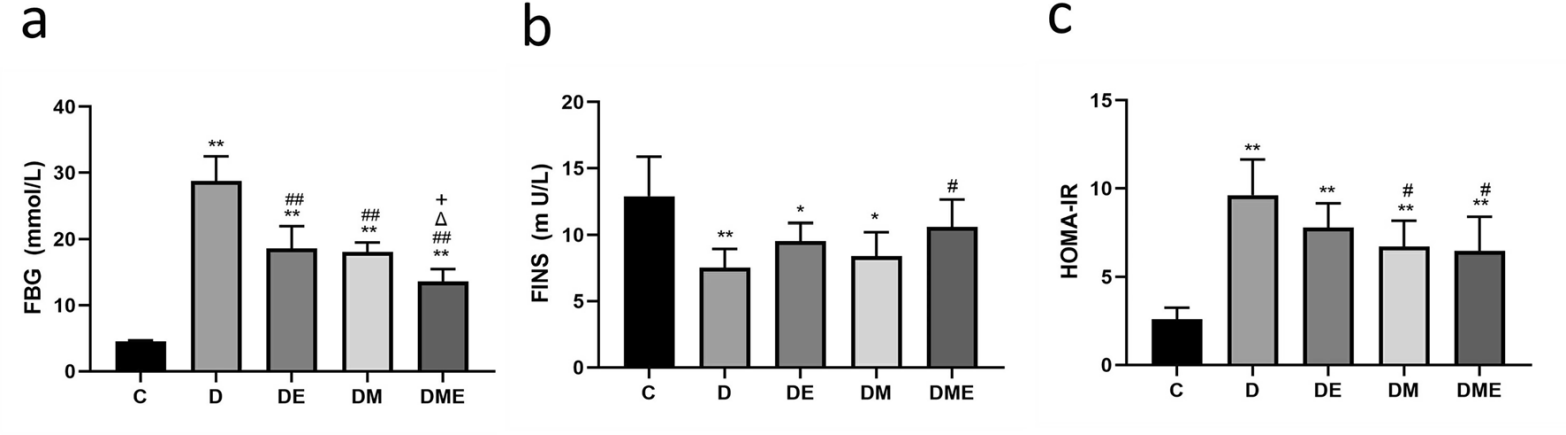
Changes of fasting blood glucose (FBG), fasting insulin (FINS) and homeostasis model assessment-estimated insulin resistance index (HOMA-IR) after 8 weeks of different interventions. (**a**) FBG is significantly lower in group DE, DM, DME than in D, and DME group is the lowest one among the diabetes rats; (**b**) FINS is higher in group DME than group D; (**c**) there were greater decreases in HOMA-IR in groups DM and DME than in group D. *p < 0.05 and **p < 0.01 compared with C; ^#^p < 0.05 and ^##^p < 0.01 compared with D; ^∆^p < 0.05 and ^∆∆^p < 0.01 compared with DE; ^+^p < 0.05 and ^++^p < 0.01 compared with DM. The number of samples was 10 in each group.

Levels of FBG and FINS were measured in T2DM rats exposed to aerobic exercise or MOTS-c intervention. Levels of FBG in group D were higher than those in group C (p < 0.01), while levels of FBG in groups DE, DM, and DME groups were lower than in group D but higher than in group C (p < 0.01), as shown in Fig. 1a. FBG levels in the DME group were the lowest in the diabetic rats (p < 0.01). The FINS index was lower in group D than in group C (p < 0.01), but higher in group DME than in group D (p < 0.05) as shown in Fig. 1b.

HOMA-IR indices in groups D, DE, DM and DME were higher than in group C (p < 0.01). There was a decrease in groups DM and DME (p < 0.05) as shown in Fig. 1c, indicating that MOTS-c reduced FBG and insulin resistance in rats with T2DM.

### Blood lipids

**Figure 2:**
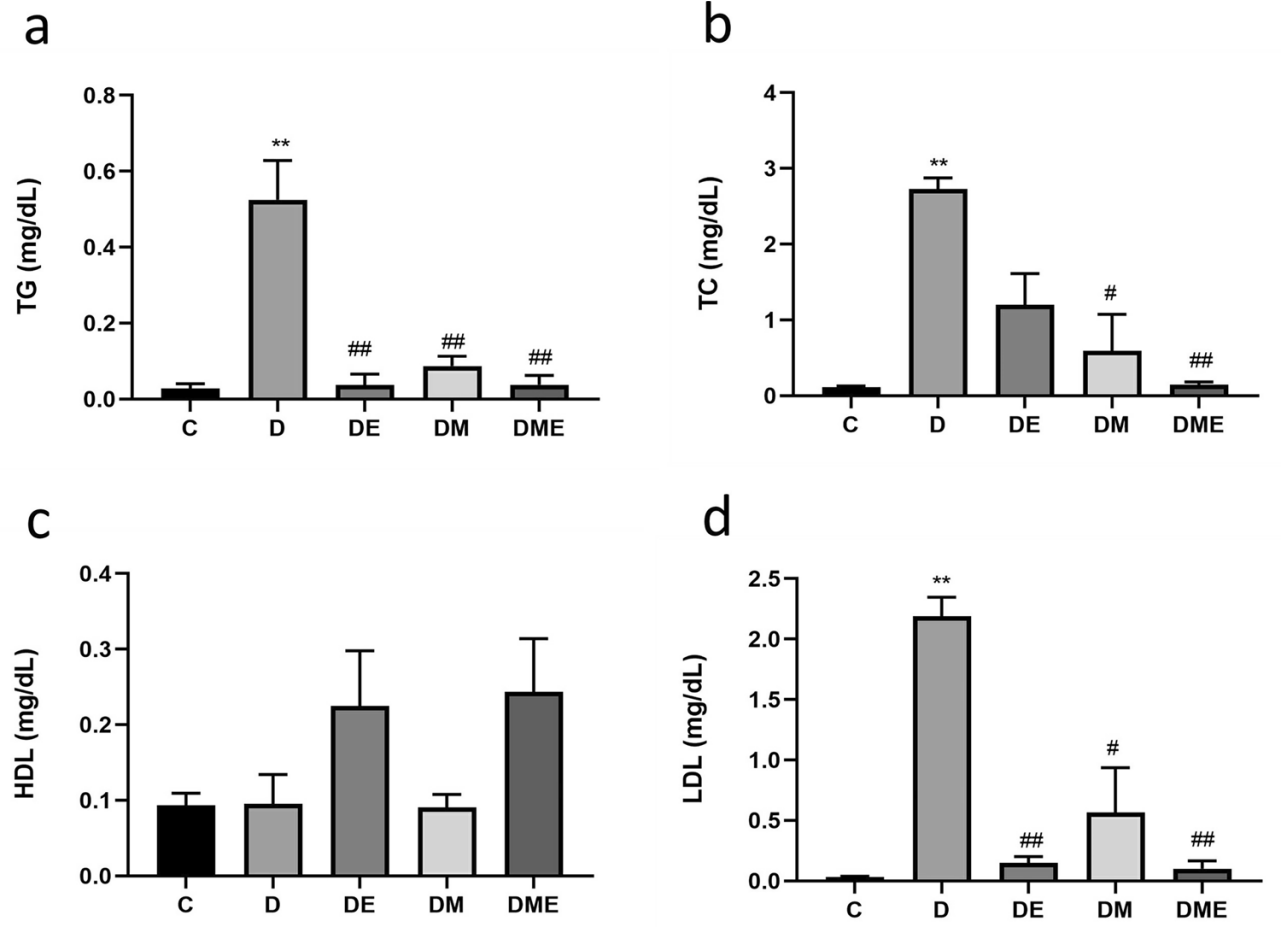
Changes of lipid levels after 8 weeks of different interventions. (**a**) Levels of Triglycerides (TG) in group D were greater than that in groups C, DE, DM and DME; (**b**) levels of total cholesterol (TC) in group D were higher than that in groups C, DM and DME; (**c**) plasma levels of high-density lipoprotein-cholesterol (HDL) were not different among groups; (**d**) low density lipoprotein-cholesterol (LDL) levels in group D were higher than that in groups C, DE, DM and DME. *p < 0.05 and **p < 0.01 compared with C; ^#^p < 0.05 and ^##^p < 0.01 compared with D; ^∆^p < 0.05 and ^∆∆^p < 0.01 compared with DE; ^+^p < 0.05 and ^++^p < 0.01 compared with DM. The number of samples was 6 in each group.

We measured the effects of eight-week aerobic exercise or MOTS-c treatment on lipid metabolism in rats with T2DM. TG Levels in group D were higher than that in groups C, DE, DM and DME (p < 0.01) (Fig. 2a). Likewise, levels of TC in group D were higher than those in groups C, DME (p < 0.01), and DM (p < 0.05) (Fig. 2b). Plasma levels of HDL were not different among groups (p > 0.05) (Fig. 2c), while LDL levels in group D were higher than those in groups C, DE, DME (p < 0.01) and group DM (p < 0.05) (Fig. 2d).

### Ultrastructural changes

**Figure 3:**
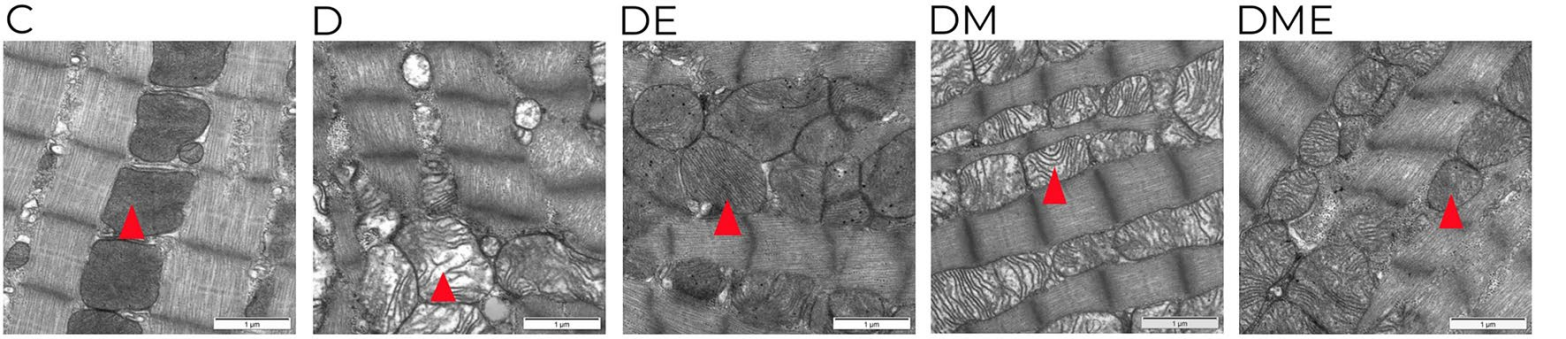
Representative images of myocardial ultra-structure ultra-images showing myocardiual fibers and mitochondria taken with a transmission electron microscope. (× 20,000 magnification, scale bar 1 μm, mitochondria denoted by arrows). Images are from the following groups: C, D, DE, DM, DME. Myocardial mitochondria from diabetic myocardium are characterized by mitochondrial swelling, loss of crest, and vacuolization. *C* control, *D* diabetic, *DE* diabetic exercise, *DM* diabetic MOTS-c treatment, *DME* diabetic exercise combined with MOTS-c treatment. The number of samples was 3 in each group.

TEM was used to examine myocardial tissue and ultrastructural changes in each group. Myocardial fibers and myocytes in group C were neatly arranged, with intact and oval-shaped mitochondria and mitochondrial cristae that were not broken or vacuolated. The myocardial fibers in group D were disordered compared to group C; the mitochondria were arranged in a disorganized manner with broken cristae and vacuolization, and the myocardial structures in groups DE, DM, and DME were restored. The mitochondrial crest was intact in group DE. The arrangement of myocardial sarcomeres in group DM was regular, and limited mitochondrial crest fractures appeared. The mitochondrial crest fractures were not seen in group DME (Fig. 3).

### Echocardiographic changes

**Figure 4:**
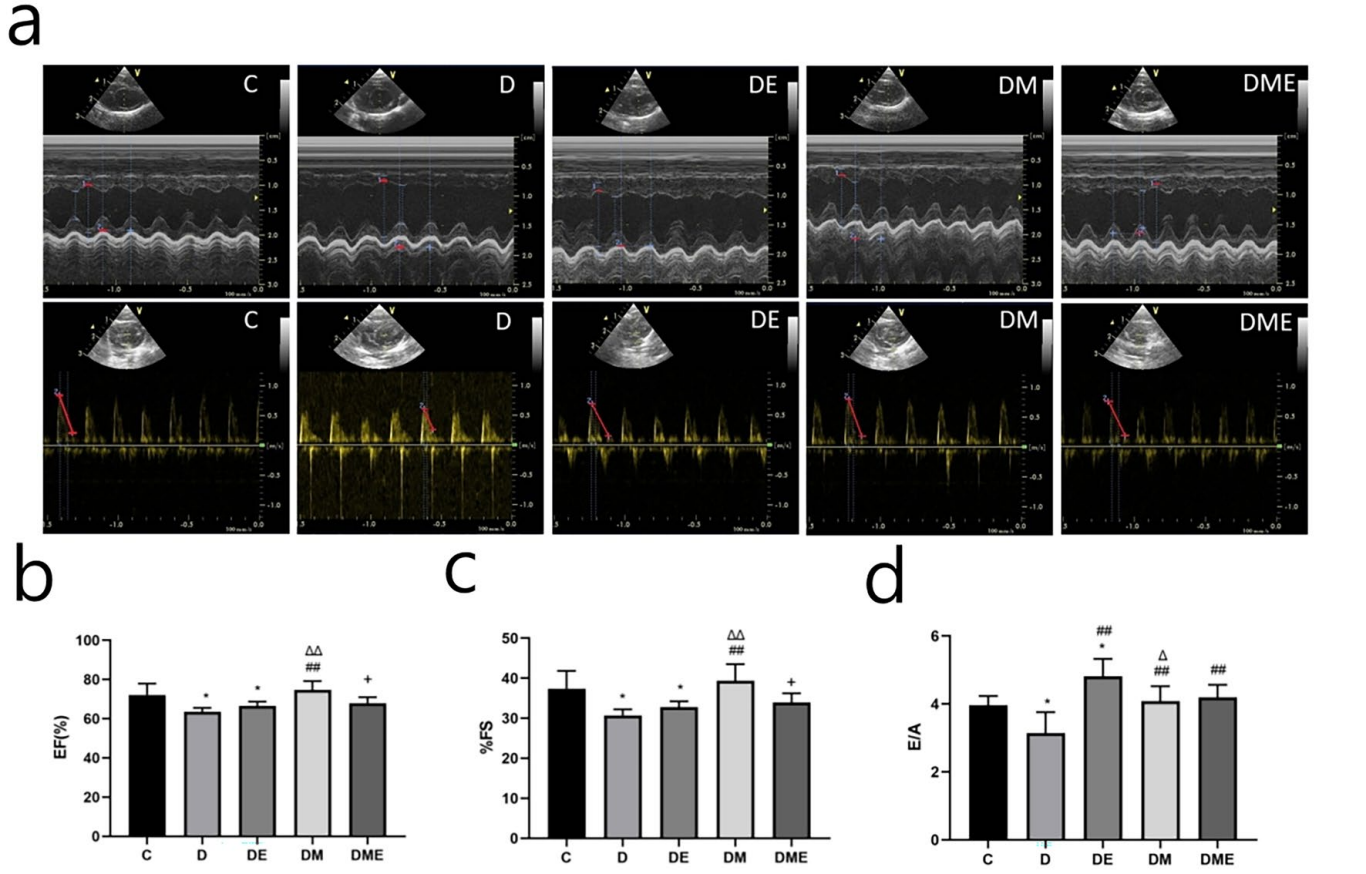
Changes of cardiac function after MOTS-c and exercise treatment. (**a**) M-mode echocardiographic images and echocardiographic doppler color flow images from rats in groups C, D, DE, DM and DME Changes of M-mode echocardiogram. Red thick lines indicating left ventricular end-diastolic dimension (LVEDD) and left ventricular end-systolic dimension (LVESD); (**b**,**c**) The ejection fraction(EF) and fraction shortening rate (%FS) in group DM were higher than those in groups D and DE; (**d**) The early diastolic filling to atrial filling velocity ratio of mitral flow(E/A) in group DE, DM and DME were higher than in group D. *p < 0.05 and **p < 0.01 compared with C; ^#^p < 0.05 and ^##^p < 0.01 compared with D; ^∆^p < 0.05 and ^∆∆^p < 0.01 compared with DE; ^+^p < 0.05 and ^++^p < 0.01 compared with DM. The number of samples was 3 in each group.

We performed echocardiographic examinations of the effects of eight-weeks of aerobic exercise or MOTS-c treatment on cardiac function in rats with T2DM (Fig. 4a)*.* Ejection fraction (EF) and left ventricular short-axis shortening (%FS), indicators of cardiac systolic function, were lower in groups D and DE compared to group C (p < 0.05). EF and %FS in the DM group were higher than those in groups D and DE (p < 0.01), while these indicators were lower in group DME than in group DM (p < 0.05) (Fig. 4b,c). These results suggest that the cardiac systolic function of diabetic rats was decreased, and that MOTS-c improved the systolic function of the diabetic heart more than aerobic exercise alone or aerobic exercise combined with MOTS-c intervention.

The mitral flow E peak to the A peak ratio (E/A), an index of the cardiac diastolic function, was lower in group D than in group C (p < 0.05), but higher in group DE than in group C (p < 0.05). The E/A values in group DE, DM, and DME were higher than in group D (p < 0.01). The E/A values in group DM were lower than those in group DE (p < 0.05) (Fig. 4d)*.* These findings indicate that the diastolic function of the heart in diabetic rats was decreased and that the positive effect of the MOTS-c intervention restored the diabetic cardiac diastolic function to near-normal levels.

### Oxidative stress

#### Changes in the biochemical indexes of myocardial oxidative stress

**Figure 5:**
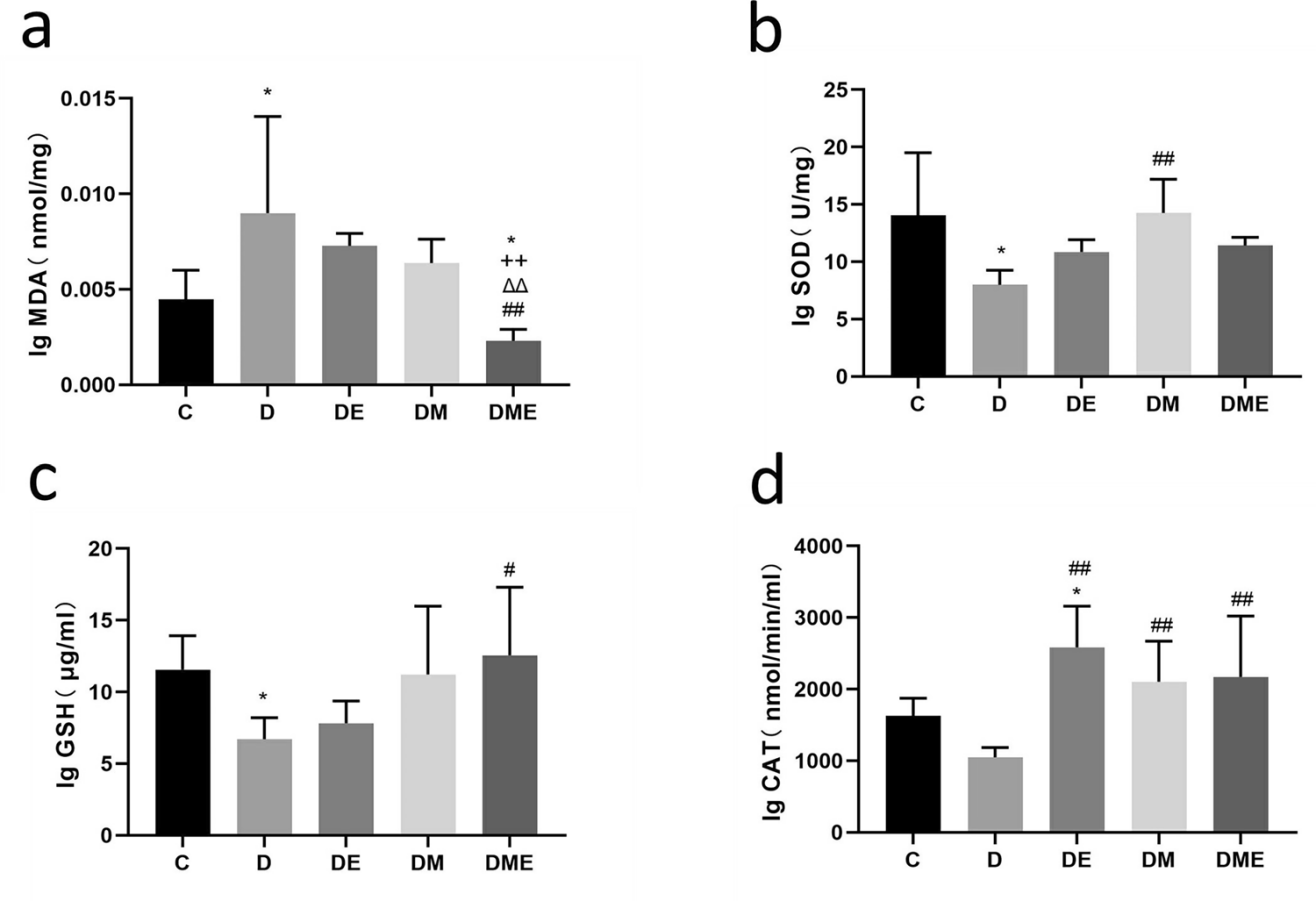
Changes of biochemical indexes of myocardial oxidative stress in rats. (**a**) lipid peroxidation Malondialdehyde (MDA) is lower in group DME than in D; (**b**) superoxide dismutase (SOD) in group DM is higher than in D; (**c**) glutathione (GSH) in group DME is higher than in D; (**d**) Catalase (CAT) in DE, DM and DME all are higher than in group D. *p < 0.05 and **p < 0.01 compared with C; ^#^p < 0.05 and ^##^p < 0.01 compared with D; ^∆^p < 0.05 and ^∆∆^p < 0.01 compared with DE; ^+^p < 0.05 and ^++^p < 0.01 compared with DM. The number of samples was 3 in each group.

Myocardial MDA levels in group D were higher than that in group C and DME (p < 0.05). Lower myocardial levels of MDA were observed in group DME than in groups D, DE, and DM (p < 0.01) (Fig. 5a). These results imply that the antioxidant effects of aerobic exercise combined with MOTS-c intervention in the myocardium of diabetic rats reduced MDA levels more than exercise intervention alone.

Levels of SOD in group D were lower than those in group C (p < 0.05) but higher in group DM than those in group D (p < 0.01). The mean value in group DM was close to that in group C, indicating that MOTS-c applied alone restored SOD levels to near normal levels (Fig. 5b).

Levels of GSH in group D were lower than those in group C and DME (p < 0.05) (Fig. 5c), suggesting that treatment with MOTS-c combined with aerobic exercise intervention caused the most significant changes in GSH levels.

Levels of CAT in groups D and C were not different (p > 0.05), while levels of CAT in group DE were higher than those in group C (p < 0.05). Also, levels of CAT in groups DE, DM and DME were higher than those in group D (p < 0.01) (Fig. 5d), which indicates a significant effect of the MOTS-c treatment or aerobic exercise or combined intervention on CAT levels in diabetic rats.

#### Myocardial MOTS-c levels

**Figure 6:**
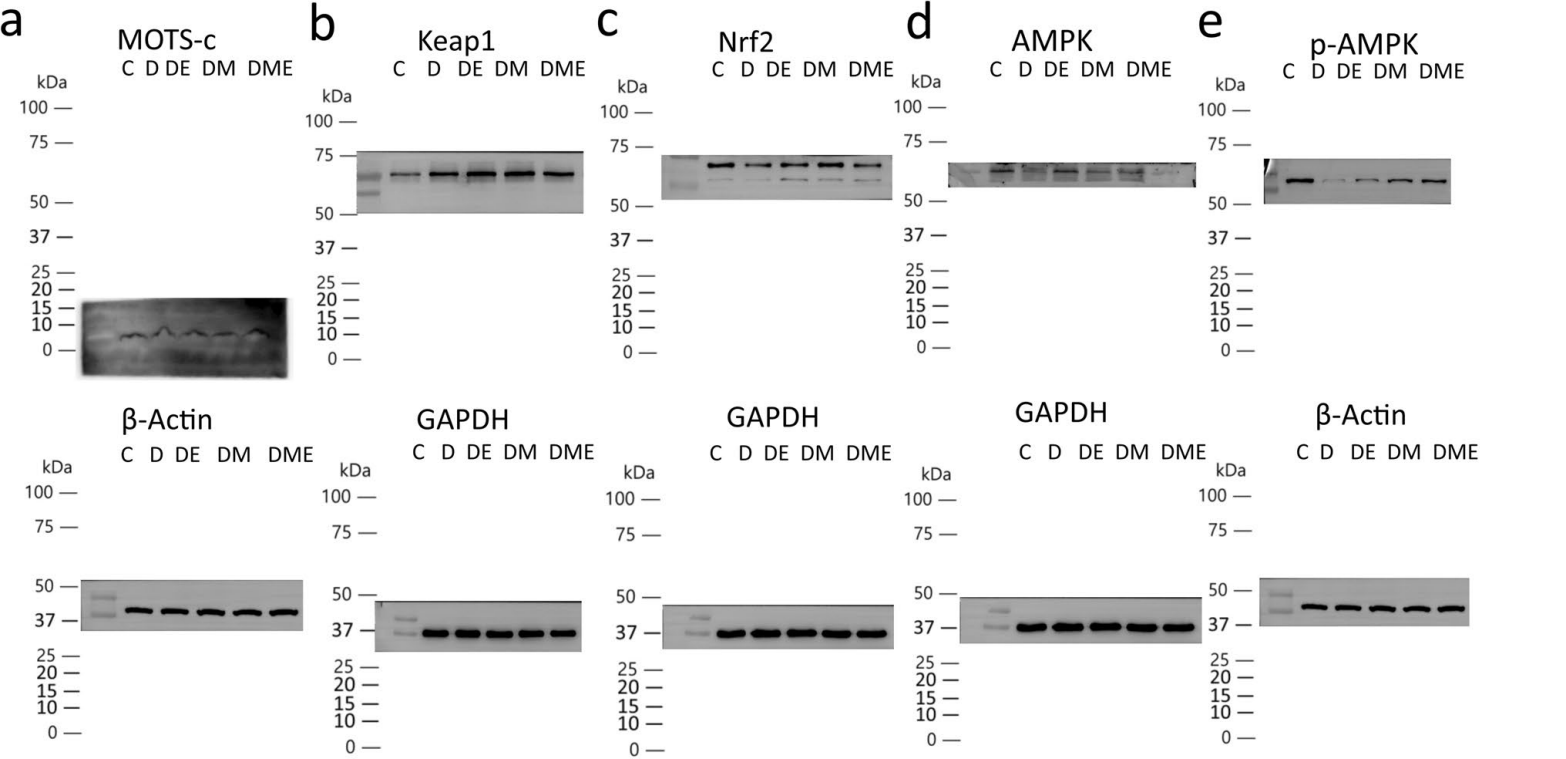
Protein expression levels of MOTS-c (**a**), Keap1 (**b**), Nrf2 (**c**), AMPK (**d**) and p-AMPK (**e**) in ventricular cardiomyocytes from rats in C, D, DE, DM and DME groups. The number of samples was 3 in each group.

**Figure 7:**
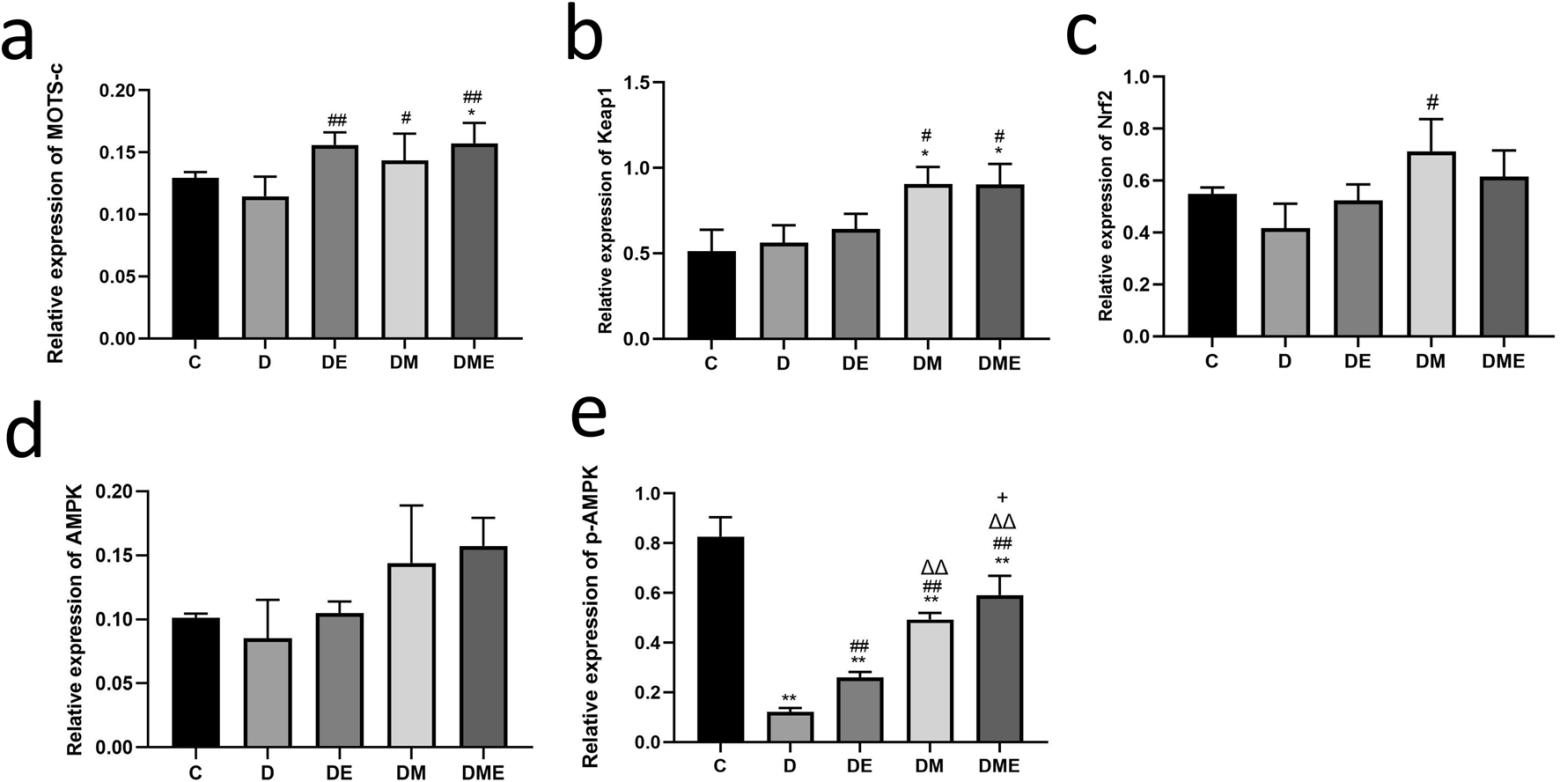
Western blot analysis of MOTS-c (**a**), Keap1 (**b**), Nrf2 (**c**), AMPK (**d**) and p-AMPK (**e**). Data are expressed as mean ± SD; *p < 0.05 and **p < 0.01 compared with C; ^#^p < 0.05 and ^##^p < 0.01 compared with D; ^∆^p < 0.05 and ^∆∆^p < 0.01 compared with DE; ^+^p < 0.05 and ^++^p < 0.01 compared with DM. The number of samples was 3 in each group.

There were no differences in myocardial MOTS-c levels in groups D and group C (p > 0.05). MOTS-c levels were higher in groups DE and DME than in group D (p < 0.01) and also higher in group DM than in D (p < 0.05). MOTS-c levels were higher in group DME than in group C (p < 0.05). Hence, the 8-week exercise alone or MOTS-c treatment with or without exercise significantly increased myocardial MOTS-c levels in diabetic rats (Figs. 6a, 7a).

#### Myocardial oxidative stress mechanism-related proteins

No significant differences in myocardial Keap1 levels were detected between rats in group D and group C (p > 0.05). The levels of Keap1 in groups DM and DME were higher than those in group C and D (p < 0.05). The results revealed that MOTS-c significantly affected myocardial Keap1 levels in diabetic rat hearts with or without aerobic exercise (Figs. 6b, 7b). Myocardial Nrf2 levels in group DM were higher than in group D (p < 0.05). These findings indicated that MOTS-c treatment increased myocardial Nrf-2 levels in in diabetic rats (Figs. 6c, 7c).

Myocardial AMPK levels did not significantly differ among the groups (p > 0.05); however, the mean values of AMPK in the DM and DME groups were higher than those in group D (Figs. 6d, 7d).

In all other groups, myocardial p-AMPK levels were lower than in group C (p < 0.01). p-AMPK levels in the DE, DM, and DME groups were higher than in group D (p < 0.01). p-AMPK levels were higher in DM and DME groups compared to group DE (p < 0.01). p-AMPK levels in the group DME were higher compared to group DM (p < 0.05). Thus, these data indicated that MOTS-c significantly affected myocardial p-AMPK levels in diabetic rat hearts with or without aerobic exercise. Exercise alone significantly affected myocardial AMPK phosphorylation, but MOTS-c was more effective than exercise intervention for myocardial p-AMPK (Figs. 6e, 7e).

## Discussion

MOTS-c is a promising therapeutic and/or prevention strategy for obesity and diabetes mellitus^42^. Herein, we used a rat model of T2DM to explore the effects of MOTS-c on the ultrastructure and function of diabetic myocardium and to observe changes in oxidative stress indicators to explore the underlying molecular mechanisms. Our most important finding was that MOTS-c mediated antioxidant defense in aerobic exercise by alleviating diabetic myocardial injury, which could be related to MOTS-c increasing the protein expression of myocardial MOTS-c, Keap1, Nrf2, and p-AMPK and antioxidant indices (SOD and CAT). MOTS-c treatment and exercise reduced myocardial lipid peroxidation levels (MDA) and increased MOTS-c, p-AMPK, and antioxidant indices (GSH and CAT).

### Glucolipid metabolism

Our study indicates that intraperitoneal injection of MOTS-c modulated fasting glucose and insulin resistance in male diabetic rats, which is also consistent with previous findings^12,13^. Simultaneous aerobic exercise and intraperitoneal administration of MOTS-c increased fatty acid oxidation and glucose utilization in skeletal muscle and reduced insulin resistance^34^. Our study also suggests that aerobic exercise combined with intraperitoneal injection of MOTS-c reduced insulin resistance. Moreover, the combined treatment had a more pronounced effect on blood glucose than exercise alone. However, 8 weeks of MOTS-c treatment did not restore fasting glucose and insulin resistance to normal levels in male diabetic rats, which may be due to the insufficient duration and treatment doses. Future studies are needed to further clarify optimal treatment conditions (Fig. 1).

While previous findings only correlated endogenous MOTS-c and lipid levels, our study complemented those findings by examining the effects of exogenous MOTS-c administration on lipid metabolism. Exogenous MOTS-c significantly improved lipid metabolism in male diabetic rats. Exercise alone and MOTS-c with or without exercise significantly affected TG and LDL in male diabetic rats. In addition, treatment with MOTS-c alone also, reduced TC levels in diabetic rats (Fig. 2).

### Cardiac structure and function

We studied the effects of MOTS-c cardiac structure and function from diabetic rats, and determined that myocardial ultrastructure in diabetic rats was restored to near-normal levels when MOTS-c was combined with aerobic exercise intervention, suggesting that MOTS-c combined with exercise intervention attenuated diabetes-induced myocardial damage (Fig. 3), as confirmed by the results of MDA testing (Fig. 5a). Regarding echocardiographic cardiac function indices, a previous study showed that diabetes or exercise training did not induce changes in ejection fraction and LV short-axis shortening, and that E/A ratios in trained diabetic animals were similar to those of sedentary controls, and that MOTS-c improved LVIDd and LVID and reduced blood pressure^43^. In our study, exercise intervention and MOTS-c treatment with or without exercise im-proved diastolic function in diabetic rats (Fig. 4d). Treatment with MOTS-c intervention also significantly improved myocardial contractile function in diabetic rats (Fig. 4b,c), as also shown in similar to the results of previous studies^32,38^. Regardless of cardiac systolic or diastolic function indices, administraion of MOTS-c alone improved diabetic cardiac function more effectively than aerobic exercise alone (Fig. 4b–d). In previous studies, AMPK signaling was considered to be associated with cardiac con-tractile function^44^. We reasoned that since both MOTS-c and exercise stimulate AMPK^32^, there may be a cumulative effect on contractile function. However, we found that the combination of exercise and MOTS-c was not more effective in improving contractile function compared to MOTS-c injection alone. A possible explanation for this unexpected result is that AMPK activation depends on tissue AMP concentration and is altered by high glucose and free fatty acid availability^45,46^. MOTS-c binding and exercise may activate AMPK rather than increase total AMPK^31^, as demonstrated by our findings (Fig. 7e). It is certain that exogenous MOTS-c administration improved insulin resistance at a systemic level and also improved cardiac function, thereby ameliorating diabetic cardiomyopathy.

### MOTS-c levels

Previous studies of circulating MOTS-c levels reported that serum MOTS-c levels are lower in patients with type 1 and type 2 diabetes^36,47^. Lower circulating MOTS-c levels are associated with insulin resistance, obesity, lipid markers TG, HDL-C, LDL-C^23^, and glycated haemoglobin^36^, as well as with gender and mitochondrial DNA (mt DNA) polymorphisms^23^. A study by Du et al. was the first to report that MOTS-c levels were associated with insulin resistance^48,49^, waist circumference, hip circumference, and waist-to-hip ratio in obese male children and adolescents^48,49^. This was also true for lipid markers such as TG, HDL-C, and LDL-C^49^. In contrast, there were no differences in MOTS-c levels of female obese children and adolescents or female mice^23,49^. In addition, the m.1382A>C polymorphism is associated with high-er circulating MOTS-c levels and influences the prevalence of T2DM in males^23^, which may be due to the correlation between mitochondrial development and sexual dimorphism in mitochondrial characteristics^49^. Plasma MOTS-c concentrations were similar in lean and obese individuals, whereas plasma MOTS-c concentrations were negatively correlated with insulin sensitivity, an effect that was better reflected in lean individuals. Therefore, the underlying mechanism of MOTS-c role in maintaining metabolic homeostasis in the early stages of insulin resistance needs to be further evaluated^48^. Circulating MOTS-c may result from tissue spillover rather than actual inter-organ communication. Induction of insulin resistance using a high-fat diet resulted in decreased skeletal muscle and circulating MOTS-c levels and reduced activity^34^. Elevated MOTS-c levels in senescent cells, which are exposed to these peptides, increased mitochondrial respiration and some senescence-associated secretory phenotypes (SASP) in the former, suggesting that mitochondria-secreted peptides exert a therapeutic effect by targeting mitochondrial energy and SASP production in senescent cells^50^. Serum MOTS-c levels are lower in patients with coronary artery disease and are negatively correlated with the severity of coronary artery disease^31^. We found no statistically significant difference in MOTS-c levels in myocardial tissue between male type 2 diabetic rats and normal rats. How-ever, the mean myocardial MOTS-c levels in diabetic rats were lower than in the control group, with a tendency to decrease, which is similar to previous findings of reduced blood^49^ or skeletal muscle MOTS-c^34^ levels in diabetic patients.

### Studies related to the effect of exercise on endogenous

MOTS-c concluded that exercise increases endogenous MOTS-c expression in the skeletal muscle and circulation in humans^51^. MOTS-c is considered an exercise response factor, as exercise increases MOTS-c production, im-proves metabolism, and promotes skeletal muscle secretion of MOTS-c in experimental animals, perhaps via the induction of skeletal muscle MOTS-c expression by synergizing the lipocalin pathway with exercise^52^. Energy control activates the AMPK/PGC-1α pathway and regulates MOTS-c expression^34^. MOTS-c, exercise, and their combination can thus regulate MOTS-c secretion and/or production in obese mice through the AMPK pathway. MOTS-c may be self-regulated, and PGC-1α upregulates MOTS-c expression through the AMPK pathway^42^. Exercise increases endogenous MOTS-c levels in the heart^12,32^, skeletal muscles^12^, and brain^12,19^. A previous study also showed that exercise or MOTS-c administration increased the MOTS-c protein content in the skeletal muscles and circulating blood in obese and in high-fat diet-induced insulin resistance state^18^. The combination of exercise and MOTS-c intervention promoted MOTS-c expression but without a superimposed effect; hence, there might be an upper limit to the increase in MOTS-c content^34^. The administraion of exogenous MOTS-c during exercise increased myocardial endogenous MOTS-c and activated AMPK^32^. Our study suggested that MOTS-c treatment alone or combined with aerobic exercise increased myocardial MOTS-c levels in diabetic rats (Fig. 7a), as also reported by others^12,32^.

### Oxidative stress

Numerous studies have reported that hyperglycemia increases ROS levels^53^, damaging myocardial tissue and vascular endothelium^1,53,54^. Nrf2 is a key transcription factor of oxidative stress^55^. Keap1 detects oxidative stress by binding of redox-sensitive cysteine residues and releases Nrf2 from Keap1-mediated inhibition^56^. Once dissociated from Keap1, Nrf2 translocates to the nucleus, where it can heterodimerize with small Maf proteins and bind to cis-acting AREs, effectively activating phase II detoxification enzymes and ameliorating oxidative stress^57^. Exercise-induced activation of Nrf2 and ROS can occur through the oxidation of cysteine residues^57^. There is much evidence that exercise increases Nrf2 levels in all tissues^58^. We observed a trend towards an increase in mean myocardial Nrf2 values in diabetic rats treated with exercise alone; however, the observed difference was not statistically significant. Although low-intensity exercise does not provide a sufficient oxidative stress stimulus to increase Nrf2 protein levels, moderate or high-intensity exercise leads to a corresponding increase in Nrf2 protein levels to maintain redox homeostasis^58^. On the other hand, regular moderate exercise training has a more significant effect on Nrf2 responses compared to strenuous exercise^57^. In our study, 8 consecutive weeks of moderate-intensity exercise combined with MOTS-c treatment significantly increased myocardial GSH, and decreased MDA levels in diabetic rats (Figs. 5, 7b).

The translocation of endogenous MOTS-c to the nucleus leads to retrograde signaling in the stressed state^59,60^. MOTS-c interacts with Nrf2, enabling Nrf2 to recognize and bind to antioxidant response elements (ARE), which initiates the transcription of target genes and directly regulates ARE-containing target genes in the nuclear genome^61,62^. Exogenous MOTS-c is dynamically transferred to the nucleus in a time-dependent manner, suggesting a direct nuclear role of MOTS-c in regulating nuclear gene expression, including its involvement in heat shock protein mediated responses and metabolism^25,51^. We previously reported that exogenous MOTS-c intervention significantly reduced myocardial ROS levels in diabetic rats^63^. Other studies reported that endogenous or exogenous MOTS-c interacts with Nrf2 through direct nuclear entry^60,62^. In contrast, our results indicated that exogenous MOTS-c counteracts oxidative stress by activating the Keap1-Nrf2 signaling pathway, which is similar to the effect of exercise to activate the Keap1-Nrf2 signaling pathway^64^. In the present study, MOTS-c intervention alone significantly increased the levels of Keap1, GSH, and CAT (Figs. 5b–d, 7b,c). Aerobic exercise combined with MOTS-c intervention reduced the levels of MDA (Fig. 5a), a marker of lipid peroxidation damage in the myocardium of diabetic rats, suggesting that MOTS-c treatment combined with exercise may attenuate oxidative stress damage in diabetic rats by decreasing myocardial lipid peroxidation levels.

It is likely that MOTS-c increases Nrf2 levels to exert protective effects against cardiac dysfunction. Previous studies reported that AMPK activation enhances Nrf2/ heme oxygenase 1(HO-1) signaling^8^. A classic study of one leg endurance training confirmed that a single bout of aerobic exercise and endurance training increased the amount and activation of AMPK^65^. Intraperitoneal injection of MOTS-c in an insulin-resistant state activated the AMPK/PGC-1αpathway, increased insulin sensitivity^42,66^, and regulated metabolic homeostasis, similar to the effect of aerobic exercise^42^. There also evidence that both endogenous and exogenous MOTS-c activates AMPK, increased phosphorylated AMPK levels, but that the total amount of MOTS-c remained unchanged. Activated AMPK regulates other proteins to improve cardiac performance^32^. Our findings indicate that exogenous MOTS-c increases levels of MOTS-c in the diabetic myocardium, in turn stimulating activation levels of AMPK (Fig. 7e), which is similar to previous findings. These results suggest that the protective influence of MOTS-c in attenuating myocardial ultrastructural damage and cardiac dysfunction in diabetic rats could be partially ascribed to the regulation pathway of the activation of AMPK activation.

### Study limitations

Our study has some limitations: we did not investigate the effects of different MOTS-C treatment regimens, e.g., related to concentrations, routes of administration and frequency of MOTS-c administration on diabetic myocardium.

## Conclusions

MOTS-c alleviates dysregulation of glucolipid metabolism and reduces myocardial structural damage and cardiac dysfunction in diabetic rats, which is partly similar to the effects of exercise. The underlying mechanisms may be associated with elevated myocardial MOTS-c levels, which in turn activates AMPK, increases Keap1 levels, and enhances Nrf2 expression. MOTS-c with exercise treatment elevates myocardial GSH, CAT, and decrease MDA levels. Therefore, MOTS-c might be used to treat oxidative stress-induced myocardial ultrastructural damage and dysfunction by activating the Keap1/Nrf2 signaling pathway; MOTS-c can mediate antioxidant defense mechanisms in aerobic exercise to reduce diabetic myocardial injury. Our findings provide experimental evidence of the molecular mechanisms of improvements in cardiac function after treatment of diabetic rats with MOTS-c, and also provides evidence of the molecular mechanisms of improvements in cardiac function after treatment of diabetic rats with MOTS-c and exercise. Our study suggests that MOTS-c may be a valuable supplement for overcoming exercise insufficiency and improving myocardial structure and function in diabetes (Supplementary Information S1).

### Supplementary Information

Supplementary Information.

## Data Availability

The data are contained within the article.
