# Supplementary material for: The role of MOTS-c-mediated antioxidant defense in aerobic exercise alleviating diabetic myocardial injury
Source: Sci Rep. 2023 Nov 13;13:19781. doi: 10.1038/s41598-023-47073-0 (PMC10643467; doi:10.1038/s41598-023-47073-0)
Supplement: Supplementary file 1 — Supplementary Information. [file 41598_2023_47073_MOESM1_ESM.pdf]

The full-length gels and blots

1. AMPK

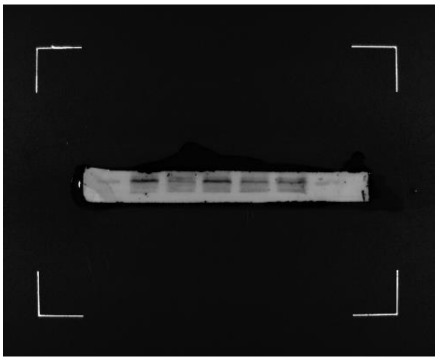

AMPK-1

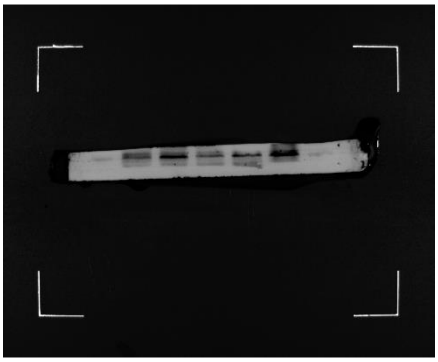

AMPK-2

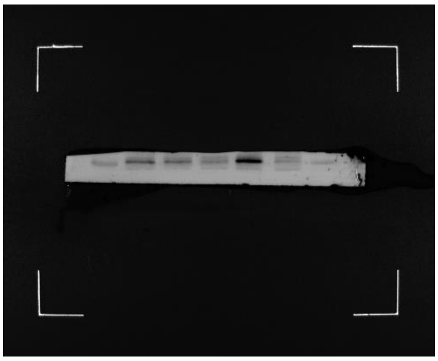

AMPK-3

2. Keap1

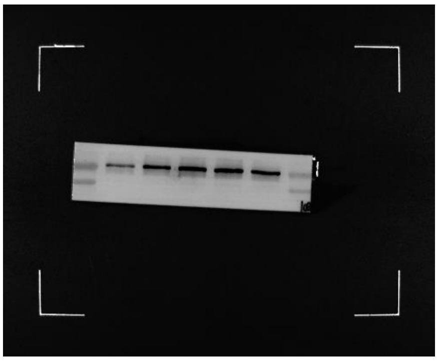

Keap1-1

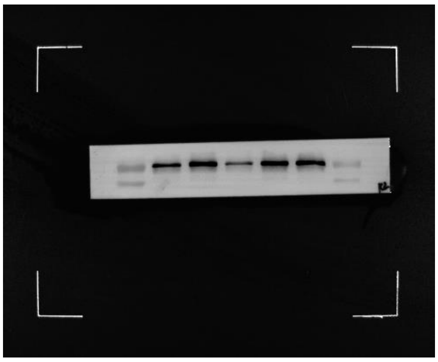

Keap1-2

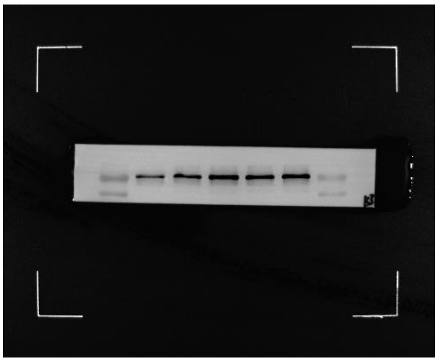

Keap1-3

3. Nrf2

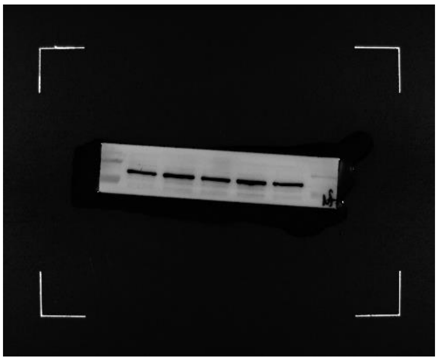

Nrf2-1

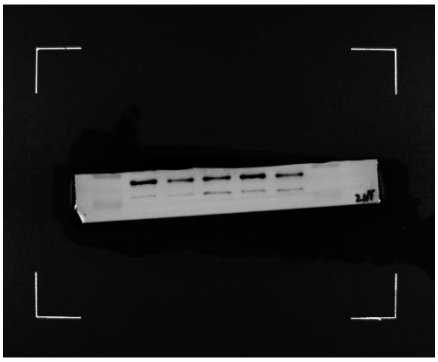

Nrf2-2

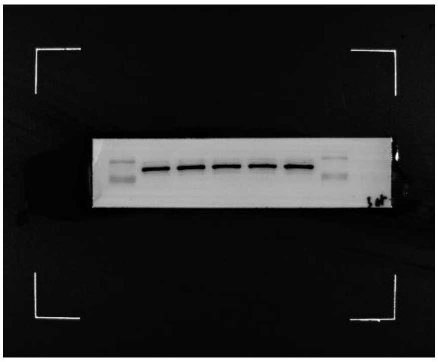

Nrf2-3

4.β-actiin

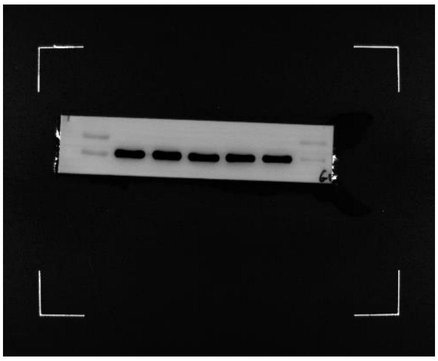

β-actiin-1

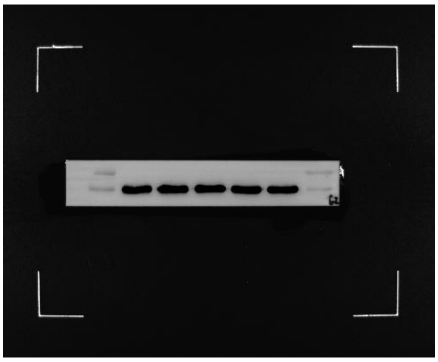

β-actiin-2

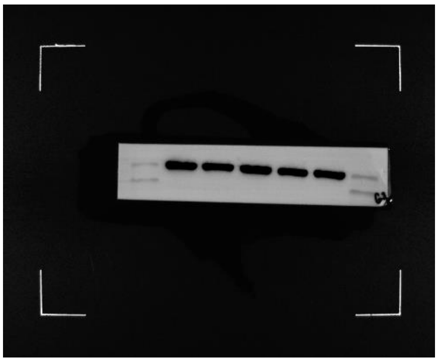

β-actiin-3

5. MOTS-c

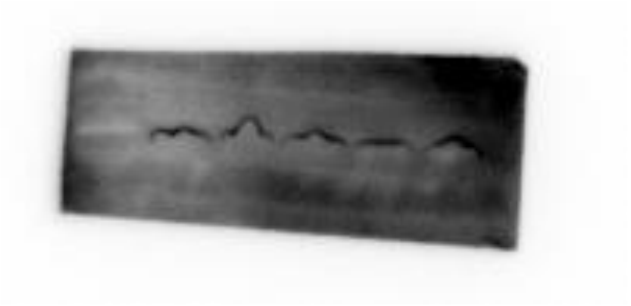

MOTS-c-1

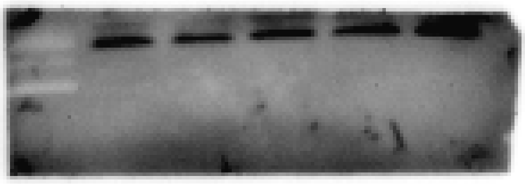

MOTS-c-2

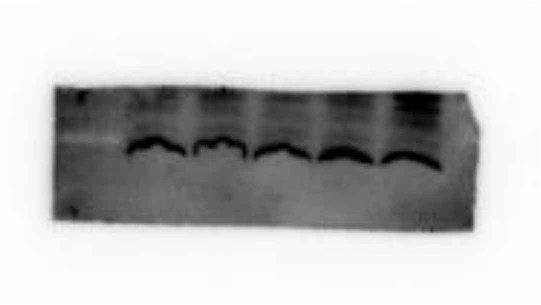

MOTS-c-3
